# Direct Interaction of Polar Scaffolding Protein Wag31 with Nucleoid-Associated Protein Rv3852 Regulates Its Polar Localization

**DOI:** 10.3390/cells10061558

**Published:** 2021-06-20

**Authors:** Rajni Garg, Chinmay Anand, Sohini Ganguly, Sandhya Rao, Rinkee Verma, Valakunja Nagaraja

**Affiliations:** 1 Department of Microbiology and Cell Biology, Indian Institute of Science, Bengaluru, Karnataka 560012, India; rajni.garg1411@gmail.com (R.G.); anandjbk2004@gmail.com (C.A.); sohiniganguly1992@gmail.com (S.G.); sandhyarao20@gmail.com (S.R.); rinkeenitk@gmail.com (R.V.); 2 Jawaharlal Nehru Centre for Advanced Scientific Research, Bengaluru, Karnataka 560064, India

**Keywords:** nucleoid-associated proteins, Mycobacterium tuberculosis, Rv3852, Wag31, DivIVA

## Abstract

Rv3852 is a unique nucleoid-associated protein (NAP) found exclusively in *Mycobacterium tuberculosis* (Mtb) and closely related species. Although annotated as H-NS, we showed previously that it is very different from H-NS in its properties and is distinct from other NAPs, anchoring to cell membrane by virtue of possessing a C-terminal transmembrane helix. Here, we investigated the role of Rv3852 in Mtb in organizing architecture or synthesis machinery of cell wall by protein–protein interaction approach. We demonstrated a direct physical interaction of Rv3852 with Wag31, an important cell shape and cell wall integrity determinant essential in Mtb. Wag31 localizes to the cell poles and possibly acts as a scaffold for cell wall synthesis proteins, resulting in polar cell growth in Mtb. Ectopic expression of Rv3852 in *M. smegmatis* resulted in its interaction with Wag31 orthologue DivIVA_Msm_. Binding of the NAP to Wag31 appears to be necessary for fine-tuning Wag31 localization to the cell poles, enabling complex cell wall synthesis in Mtb. In Rv3852 knockout background, Wag31 is mislocalized resulting in disturbed nascent peptidoglycan synthesis, suggesting that the NAP acts as a driver for localization of Wag31 to the cell poles. While this novel association between these two proteins presents one of the mechanisms to structure the elaborate multi-layered cell envelope of Mtb, it also exemplifies a new function for a NAP in mycobacteria.

## 1. Introduction

Lack of a nuclear membrane presents bacteria with an exigent task of compacting their genome in a small cytoplasmic volume. The highly basic proteins, known as the nucleoid-associated proteins (NAPs), are associated with the nucleoid, enabling it to segregate, replicate, and transcribe in a manner appropriate to the needs of the bacterium [1]. NAPs play a crucial role in maintaining the spatio-temporal organization of the nucleoid and influence gene expression through topological changes such as coating, bending, wrapping, and bridging DNA [2,3].

Genome maintenance and DNA transactions are orchestrated by only a few NAPs in *Mycobacterium tuberculosis* (Mtb), as opposed to *Escherichia coli* (*E. coli*) and other well-studied bacteria that possess more than a dozen NAPs. Different mycobacterial NAPs can act as mediators for different forms of intracellular stress. Lsr2, a mycobacteria-specific NAP, is upregulated in nutrient starvation [4], hypoxia, and antibiotic exposure [5], while GroEL1 is upregulated in heat shock [6], low-aeration [7], and oxidative stress conditions [8] and macrophage infections [9]. Rv3852 is a NAP that is present only in slow-growing species of mycobacteria [10]. It has KAAK PAAK repeats similar to those found in MtHU and H1/H5 family histones [10,11]. It was annotated as a possible H-NS [12]. However, our earlier studies showed that Rv3852 could not complement *E.coli* H-NS mutant and also did not resemble H-NS in any of its properties [10]. Unlike any other NAP, a small stretch of hydrophobic amino acids located in the C-terminal of the protein tethers the protein to the cell membrane, while the protein is still bound to the DNA [10], resulting in a more diffused nucleoid. Notably, the deletion of C-terminal helix leads to loss of membrane tethering, and the deletant (Rv3852∆ctd) behaved like a true NAP by condensing the nucleoid [10]. The unusual localization of Rv3852 prompted us to investigate its role in Mtb physiology through protein–protein interaction approach. Here, we present data of direct physical interaction of Rv3852 with Wag31, an important cell wall integrity determinant in Mtb.

Wag31 is a homologue of *Mycobacterium smegmatis* DivIVA [13]. Unlike the restricted presence of Rv3852, DivIVA is found in a diverse group of bacteria [13,14,15]. Although it is conserved across species, the homologues function in a species-specific manner exhibiting diverse roles [13,16,17,18,19]. In vegetative *Bacillis subtilis*, DivIVA interacts with MinC and MinD, retaining them at the cell poles in order to ensure division septum can form at the mid-cell [18,19,20]. However, during sporulation, DivIVA has two functions. First, it interacts with RacA, anchoring the chromosome at the poles [21]. Second, by binding to Spo0J, it participates in chromosome segregation [21,22,23]. However, despite its multiple roles, DivIVA is not essential for *B. subtilis*. But it has an essential function in *S. coelicolor*, where it localizes at hyphal tips and leads to polarized growth by interacting with Scy protein [24]. In other actinobacteria, DivIVA is required for cell shape maintenance [13], tethering of origin of replication [25], protection against oxidative stress [26], and co-localization with cell wall biosynthetic proteins at the pole [27,28]. Similarly, DivIVA has many roles in *Listeria monocytogenes*, including secretion of autolysins [17], division septum positioning [16], and swarming motility [29]. In *M. smegmatis*, DivIVA interacts with ParA, the chromosome partitioning ATPase, and this interaction regulates the cell elongation rate [30,31]. In Mtb, Wag31 is essential, localizing to the cell poles to interact with enzymes involved in initial steps of synthesis of cell wall precursors [27]. Here, we show that Rv3852 interacts with Wag31, and this interaction facilitates Wag31 localization to the cell poles and the cell wall, thus enabling nascent peptidoglycan synthesis.

## 2. Materials and Methods

### 2.1. Bacterial Strains and Media

*Mtb* H37Rv and H37Ra strains were grown on Middlebrook 7H11 agar (Difco; BD Bioscience, Franklin Lakes, NJ, USA) containing 10% (*v*/*v*) oleic acid, albumin, dextrose, catalase (OADC), 0.5% (*v*/*v*) glycerol or in Middlebrook 7H9 broth (Difco; BD Bioscience, Franklin Lakes, NJ, USA) supplemented with 10% (*v*/*v*) albumin, dextrose, catalase (ADC), 0.2% (*v*/*v*) glycerol, and 0.05% (*v*/*v*) Tween 80, at 37 °C with shaking at 180 rpm. *M. smegmatis* strains were grown in Middlebrook 7H11 agar or in 7H9 broth at 37 °C with shaking at 160 rpm. For the Mtb growth curve experiment, Sauton’s medium (Himedia Laboratories, India) was used. Hygromycin and kanamycin were used at concentrations of 50 µg mL^−1^ and 25 µg mL^−1^, respectively, for *Mycobacterium* strains. *E. coli* DH5α was grown in Luria–Bertani (LB) medium. Kanamycin (50 µg mL^−1^) and hygromycin (150 µg mL^−1^) were used for various recombinant *E. coli* DH5α strains.

### 2.2. Co-Immunoprecipitation of Rv3852 Interacting Proteins from Mtb and M. smegmatis

Mtb H37Ra and MsRv3852 strains were grown until mid-exponential phase, and co-immunoprecipitation was carried out as described previously [32,33]. Elutions of the antibody protein A sepharose beads and the only protein A control beads were resolved on SDS-PAG until 1cm migration, and the sample containing pool of proteins was excised. Subsequently, the samples were carbamidomethylated and trypsinized. The tryptic digests were injected on a Dionexnano-LC system followed by MS/MS analysis on a Bruker Maxis Impact QTOF instrument. The spectra obtained were analyzed using Mascot search. The unique protein hits present in the experimental and absent in control samples were considered putative interactors. For confirmation of interaction in Mtb H37Rv, the lysate of exponential phase culture was prepared by bead beating (Fast Prep Instrument, MP Biomedicals; 6.0 m/s speed; 30 s on/30 s off) in a buffer containing 10 mM Tris (pH 7.4), 100 mM NaCl, and 10 mM PMSF. The lysate was treated with 10 U each of DNase I and RNase A. A total of 2.5 mg lysate was incubated with 6× His-tagged Rv3852 protein for 2 h at 4 °C and then incubated with Ni-NTA beads for 2 h and centrifuged. The interacting proteins were eluted, electrophoresed on a 12% SDS-polyacrylamide gel and transferred onto PVDF membrane. The membrane was cut at 25 kDa, and the lower blot was incubated with 1:10,000 dilution of anti-His antibody (Abcam, UK) in 1X Tris-buffered saline (TBS) with 2% BSA (MP Biomedicals, Santa Ana, CA, USA) for 2 h, followed by three 10 min washes with TBST (TBS with 0.1% Tween 20). Later, it was incubated with 1:10,000 dilution of HRP-conjugated anti-mice secondary antibody (Abcam, UK) in TBS with 2% BSA for 2 h. Similarly, the upper blot was incubated with 1:5000 diluted Wag31 antibody, washed, and then incubated with 1:10,000 dilution of HRP-conjugated anti-rabbit antibody. The blots were developed with chemiluminescent HRP substrate (Merck Millipore, Burlington, MA, USA) using Image quant LAS 4000 system (GE Healthcare, Chicago, IL, USA).

### 2.3. Generation of M. smegmatis and Mtb Strains

The coding DNA sequences corresponding to Rv3852 and Rv3852∆ctd were PCR-amplified from pJ-Rv3852 plasmid [10] using primer sets 3852pMV261FP, 3852pMV261RP, and 3852pMV261FP, and 3852dctdpMV261RP, respectively (Table S1). The amplicons were digested with BamHI and HindIII enzymes and ligated in BamHI/HindIII-digested pMV261 vector to generate plasmids pM-Rv3852 and pM-Rv3852∆ctd. These were then transformed in *M. smegmatis* to generate MsRv3852 and MsRv3852∆ctd strains, respectively. Mtb Rv3852KO was obtained from Carl F. Nathan, Weill Cornell Medicine, New York. pM-Rv3852 and pM-Rv3852∆ctd plasmids were transformed in Rv3852KO to generate Rv3852KO/Rv3852 comp and Rv3852KO/Rv3852∆ctdcomp. The conditional knockdown of Wag31 (Wag31CKD) was generated by CRISPR-Cas9 approach [34]. Two small guide RNA (sgRNA) targeting the non-template strand of *wag31* gene, namely, sg1 (35 bp upstream of translational start site) and sg2 (24 bp downstream of translational start site), were cloned in pRH2521 vector and transformed in Mtb H37Rv-dcas9 to generate two separate strains (Table S1). The colonies obtained after transformation of each gRNA were grown and screened for decrease in expression of Wag31 protein upon addition of 600 ng/mL anhydrotetracycline (ATc) (Sigma-Aldrich, Saint Louis, MO, USA) by Western blotting. One of the colonies obtained after transformation of sgRNA2 was used as Wag31CKD for further experiments.

### 2.4. Purification of Proteins

Rv3852 and Rv3852∆ctd proteins were purified as described previously [10]. Wag31-pet28a and Wag31(96-234)-pet28a constructs were obtained from Stewart Cole, Pasteur Institute, Paris. Wag31 protein was purified from *E. coli* BL21 cells transformed with Wag31-pet28a by urea denaturation and on-column renaturation, as described previously [35]. Wag31(96-234)-pet28a containing 96 to 234 residues of Wag31 (Wag31∆N) was transformed in *E. coli* BL21 cells. Wag31∆N protein was purified from these cells post-induction at 0.5 OD_600_ with 0.5 mM IPTG for 6 h. The cultures were harvested, resuspended in lysis buffer (10 mM Tris (pH 8.0), 250 mM NaCl, and 0.5 mM PMSF) and sonicated. The sonicate was centrifuged at 29,000 rpm for 1 h at 4 °C. The supernatant collected was incubated with Ni-NTA resin and pre-equilibrated with lysis buffer for 2 h. The protein was eluted with elution buffer containing 10 mM Tris (pH 8.0), 100 mM NaCl, 400 mM imidazole, and 0.5 PMSF. For purification of Rv3852-MBP and Rv3852∆ctd-MBP, *E. coli* C41 cells harboring these plasmids were grown until 0.5 OD_600_ and induced with 0.5 mM IPTG for 5 h. The cells were harvested, resuspended in column buffer (10 mM Tris (pH 7.4), 300 mM NaCl, 10% glycerol, and 10 mM DTT with protease inhibitor tablet) and sonicated. The sonicate was centrifuged at 29,000 rpm for 1 h at 4 °C. The supernatant was incubated with amylose beads (NEB, Ispwich, MA, USA) pre-equilibrated with column buffer for 2 h. The beads were washed with column buffer and the proteins were eluted with column buffer containing 50 mM maltose. The elutes containing pure protein were pooled, dialyzed, concentrated, and stored at −80 °C until further use.

### 2.5. In Vitro Protein–Protein Interaction

For testing interaction of Wag31 with variants of Rv3852, 1 µg of Rv3852 and Wag31 proteins were incubated in 1× Co-IP buffer (10 mM Tris pH 7.4, 100 mM NaCl) for 15 min at 4 °C and pre-blocked, and anti-Wag31 antibody-coated Protein A sepharose beads were added and incubated for 2 h. The beads were sedimented by centrifugation. The bound proteins were eluted, electrophoresed on 12% SDS-polyacrylamide gel, and silver stained. The reaction mixtures containing only Wag31 and only Rv3852 protein were used as positive and negative controls, respectively. Similar reactions were carried out for Wag31 and Rv3852∆ctd also. For testing the interaction of Rv3852-MBP with Wag31, we incubated 1 μg Rv3852-MBP and Wag31 proteins for 15 min at 4 °C, and subsequently incubated them with amylose beads in 1X Co-IP buffer (10 mM Tris (pH 7.4), 100 mM NaCl) for 2 h. The mixture was centrifuged, and the proteins bound to the beads were eluted, electrophoresed on 12% SDS-PAG, and silver stained. Similarly, interaction experiments were carried out for Rv3852-MBP and Wag31∆N. Only MBP protein was used as negative control. The affinity precipitation reactions were also carried out for Rv3852∆ctd-MBP, Wag31, and Wag31∆N.

### 2.6. Growth Curves

The primary cultures of Mtb WT, Rv3852KO, Rv3852KO/Rv3852 comp, and Rv3852KO/∆ctd comp were grown until 1.0 OD_600_. The secondary cultures were inoculated in 50 mL Sauton’s media (Himedia Laboratories, India) in conical screw-capped flasks, such that initial OD_600_ was 0.02. The OD_600_ values were measured after every 48 h for 15 days. The *M. smegmatis* cultures were grown in 7H9 media, and OD_600_ values were measured after every six hours for 2 days. The mean values from three independent experiments were plotted using Graph Pad Prism 6.0 software. The significance values were calculated by using unpaired Student’s *t*-test in MS excel.

### 2.7. Sub-Cellular Fractionation

Five hundred milliliter cultures of Mtb H37Rv, Rv3852KO, and Wag31CKD were grown until 0.2 OD_600_; 600 ng/mL ATc was added for induction; and the cultures were harvested at 0.8 OD_600_. The cells were fixed with 4% paraformaldehyde (PFA) and treated with UV for 30 min, and then sub-cellular fractionation was performed as described previously [10,36]. Briefly, the cell pellet was washed; resuspended in buffer containing 10 mM Tris (pH 7.4), 100 mM NaCl, and protease inhibitor tablet; and disrupted using French pressure cell and sonication. The unbroken cells were removed by low-speed centrifugation at 8000× *g* for 20 min, and the supernatant was centrifuged at 27,000× *g* for 40 min to retrieve cell walls. The supernatant was subjected to high-speed centrifugation at 100,000× *g* for 1 h to obtain cell membranes in the pellet and the cytosol in the supernatant. Total protein was extracted from the cell membrane and cell wall by washing and resuspending them in 0.1 g n-Dodecyl β-D-maltoside (DDM)/g (Sigma-Aldrich, Saint Louis, MO, USA) of cell wall or membrane at 4 °C for 2 h. The protein concentration was measured using Bradford’s reagent. A total of 100 μg of each fraction was separated on two 12% SDS-polyacrylamide gels and transferred to activated PVDF membrane, and then immunoblotting was performed. Rabbit-raised Rv3852 and Wag31 primary antibodies (1:5000) were used as primary antibodies for each blot, and anti-rabbit HRP-conjugated secondary antibody (1:10,000) was used as secondary for both the blots. Anti-Wag31 antibody was used for probing DivIVA_Msm_ in *M. smegmatis* cell fractions. The blots were developed with chemiluminescent HRP substrate (Merck Millipore, Burlington, MA, USA) using Image quant LAS 4000 system (GE Healthcare, Chicago, IL, USA).

### 2.8. Immunofluorescence

Exponential phase cultures of Mtb H37Rv, Rv3852KO, Rv3852KO/Rv3852 comp, and Rv3852KO/∆ctd comp were harvested and washed twice with 1× PBS. The cultures were fixed for 30 min using 4% PFA, washed, and treated with 2 mg/mL lysozyme for 10 min for permeabilization. The cells were blocked with 1% FBS and incubated with 1:200 dilutions of anti-Wag31 antibody (raised in rabbit) and anti-Rv3852 antibody (mouse-raised) overnight at 4 °C. The cells were subsequently incubated with 1:250 dilutions of anti-rabbit Alexa-fluor 543 and anti-mouse Alexa-flour 488 antibodies (Thermo Fisher Scientific, Waltham, MA, USA). The nucleoids were stained with 10 µg mL^−1^ DAPI (Sigma -Aldrich, Saint Louis, MO, USA) for 30 min, mounted with prolong glass antifade (Thermo Fisher scientific, Waltham, MA, USA), and visualized under 100× objective with 2× zoom using Zeiss LSM-880 microscope. The *M. smegmatis* strains were stained using same protocol, except that anti-rabbit Alexa-fluor 647 (Thermo Fisher Scientific, Waltham, MA, USA) was used. *M. smegmatis* strains were visualized under Zeiss LSM-710 microscope. The images for both Mtb and *M. smegmatis* were processed with Zen blue 2.3 lite software. Using profile tool of the software, a line was drawn along the long axis of bacterium, and the fluorescence intensities for both the proteins were recorded along the length of each bacterium. The bacterial length was normalized to 1. The percent fluorescence intensity with respect to the maximum fluorescence intensity for each protein was plotted along the length of bacteria using MS excel.

### 2.9. BODIPY FL Vancomycin Staining

The pattern of nascent peptidoglycan synthesis was visualized in Mtb and *M. smegmatis* strains by using a protocol published earlier [37]. The Mtb strains were grown until 0.4 OD, and 1 mL culture was incubated with 1 µg mL^−1^ BODIPY FL Vancomycin (Thermo Fisher Scientific, Waltham, MA, USA) and grown for 16 h. The cells were harvested, washed with 1X PBS, fixed with 4% PFA and stained with 10 µg mL^−1^ DAPI (Sigma-Aldrich, Saint Louis, MO, USA) for 30 min, mounted with prolong glass antifade (Thermo Fisher Scientific, Waltham, MA, USA), and visualized under 100X objective with 2X zoom using Zeiss LSM-880 microscope. The images were processed with Zen blue 2.3 lite software, and the percent fluorescence intensity along the length of the bacteria was plotted as described above. The same protocol was followed for staining *M. smegmatis* strains, except the time of staining was 3 h. 

## 3. Results

### 3.1. Rv3852 Interacts with Wag31 in Mtb

**Figure 1:**
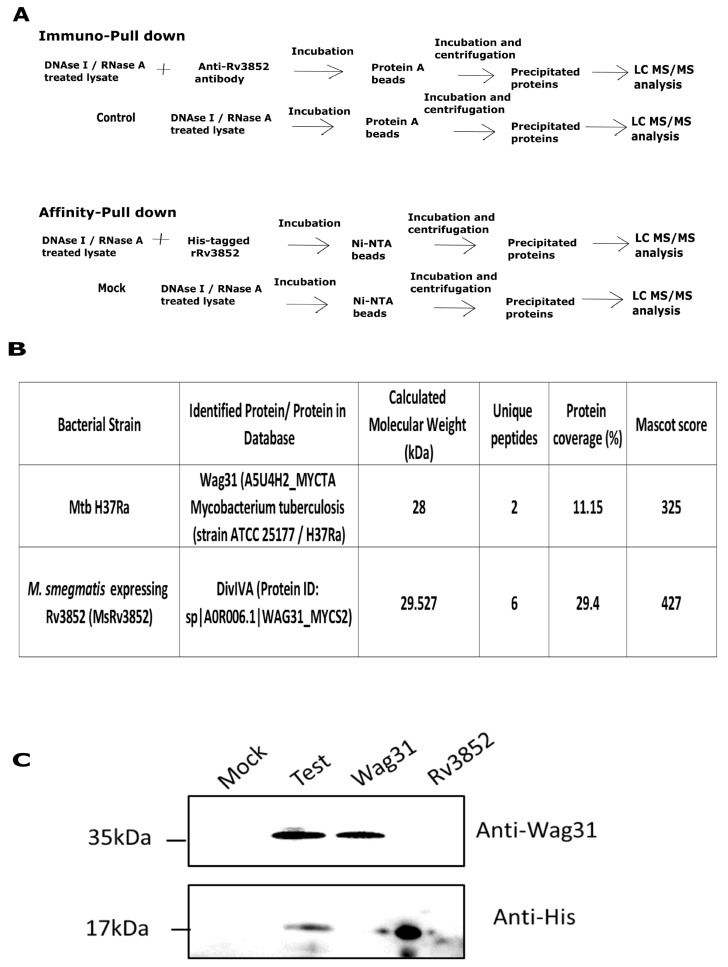
Identification of Wag31 as an interacting partner of Rv3852 in Mtb. (**A**) Schematic representation of immuno pull-down and affinity pull-down strategy. DNase I and RNase A-treated lysate was incubated with anti-Rv3852 antibody-coated protein A beads (immuno pull-down) or hexa-histidine tagged, Rv3852-coated Ni-NTA beads (affinity pull-down). The subsequent steps were carried out as indicated. Wag31 and DivIVA_Msm_ were immuno-precipitated from Mtb H37Ra and MsRv3852 lysates, respectively, using immuno pull-down. (**B**) Table shows the number of peptides, protein coverage, and mascot score for Wag31 and DivIVA_Msm_ obtained from nano-LC–MS/MS analysis from both the samples. (**C**) Immunoblot showing the interaction of hexa-histidine-tagged Rv3852 with Wag31, seen by affinity pull-down strategy outlined in (A). Mock reaction contains non-spiked lysate, while test reaction contains lysate spiked with hexa-histidine-tagged Rv3852. Wag31 and Rv3852 lanes show respective purified proteins.

The unusual membrane localization of Rv3852 suggested an unusual function for the NAP in biology of Mtb. One way to approach the problem is to identify its interacting partners. Thus, to investigate its role by finding out its partner proteins, we employed two strategies of protein–protein interaction, namely, immuno pull-down and affinity pull-down (Figure 1A). In immuno pull-down (IP) experiments carried out in Mtb H37Ra lysates using anti-Rv3852 antibodies, Wag31 was found to be an interacting partner of Rv3852, which was absent in only beads control (Figure 1B). Wag31 is an essential cell wall elongation protein in Mtb [13,38] and is conserved in mycobacteria as well as Gram-positive bacteria. Notably, Rv3852 also interacted with DivIVA_Msm_, which is an orthologue of Wag31, when it was ectopically expressed with *M. smegmatis* (MsRv3852 strain; Figure 1B). Being an orthologue, DivIVA_Msm_ has high sequence similarity (85%) with Wag31, performs a similar function in *M. smegmatis*, and is often termed as Wag31. To confirm the interaction in Mtb H37Rv, the whole cell lysate was spiked with 6X His-tagged Rv3852 protein, and the interacting partners were precipitated using Ni-NTA beads. While both the proteins were observed in the test sample, the two proteins were not detected in the mock sample (non-spiked lysates), confirming an interaction between these two proteins (Figure 1C). Thus, in all the three pull down strategies employed including the one with *M. smegmatis* lysates, Rv3852 was found to be the interacting partner of Wag31. In our other pull-down strategies with various NAPs (HU, Lsr2, Rv0430) in unrelated projects (data not shown), Wag31 was not found to be an interacting partner, indicating the specific interaction between these two proteins. All these observations together substantiate the specific interaction between an Mtb-specific NAP and a cell pole-localizing protein.

### 3.2. Direct Physical Interaction between Rv3852 and Wag31

**Figure 2:**
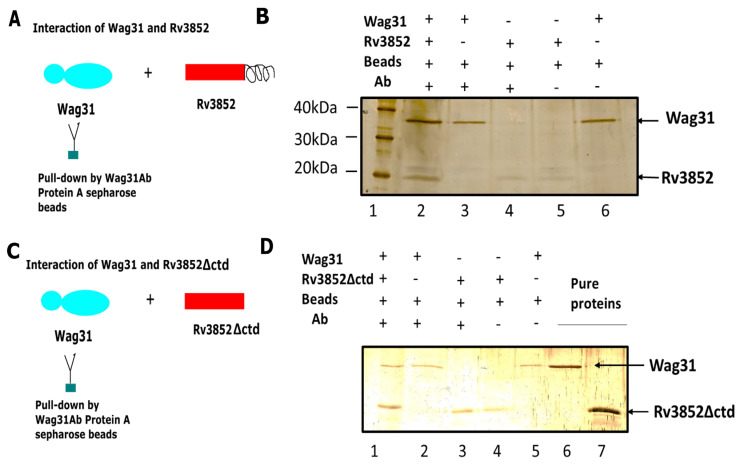
Direct physical interaction between Rv3852 and Wag31. The direct physical interaction was tested between Rv3852 and Wag31 by setting up reactions according to the scheme outlined in (**A**). (**B**) The silver-stained SDS-PAGE of pellet fraction obtained after pull-down with Wag31 antibody-coated beads. Immuno-precipitated Rv3852 is shown in the test lane (lane 2), and the positive (Wag31, Ab, beads), protein control, and bead controls for both the proteins are shown in lanes 3, 4, 5, and 6. (**C**) The interaction experiment between Wag31 and Rv3852∆ctd was set up as shown. **(D)** SDS-PAGE showing interaction between Rv3852∆ctd and Wag31. Immuno-precipitated Rv3852∆ctd is shown in the test lane (lane 1). The positive, protein, and bead controls with both the proteins are shown in lanes 2, 3, 4, and 5. The pure proteins are shown in lanes 6 and 7. The contents of each reaction are shown on top of gels in (B) and (D).

To verify whether Rv3852 interacts directly with Wag31 and not through another mediator protein in the lysate, we carried out the following experiment. Immunoprecipitation reactions were carried out using anti-Wag31 antibody-coated protein A sepharose beads in a reaction mixture containing equal concentrations of both recombinant proteins. The reaction schemes are shown in Figure 2. Both Wag31 and Rv3852 proteins were observed in the test sample (lane 1; Figure 2B). As both Rv3852 [10] and Wag31 [39] are membrane-localized, we investigated whether the C-terminal transmembrane region of Rv3852 is responsible for the interaction with Wag31. In immunoprecipitation reactions similar to the ones described above, both Rv3852∆ctd and Wag31 were recovered in the pellet fraction (lane1; Figure 2D), suggesting that C-terminal region is not involved in the interaction of Rv3852 with Wag31 protein.

### 3.3. Rv3852 Interacts with N-terminal Region of Wag31

**Figure 3:**
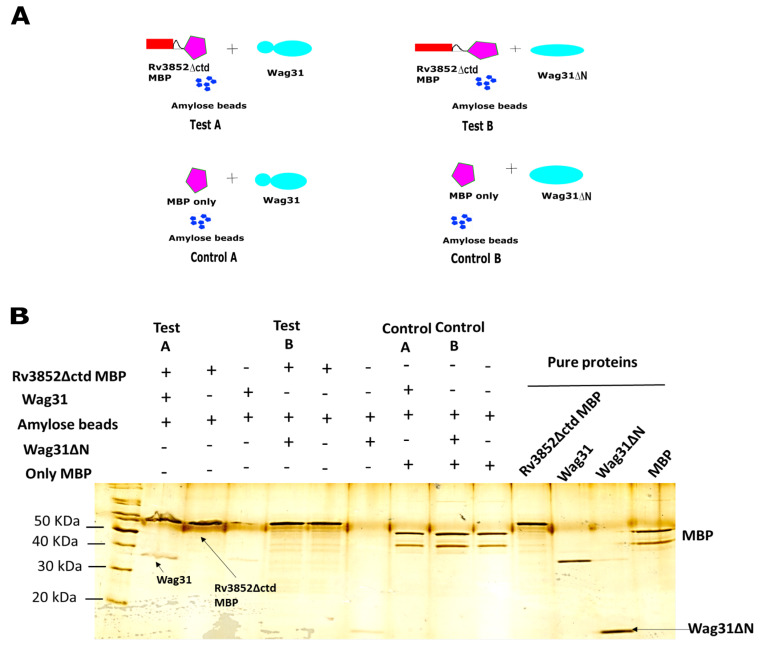
Rv3852∆ctd interacts with N-terminal region of Wag31. The co-immunoprecipitation reactions using amylose beads were carried out as indicated in (**A**). The test reactions A and B contain Rv3852∆ctd-MBP with Wag31 and Rv3852∆ctd-MBP with Wag31∆N, respectively. The control pairs A and B show negative control reactions containing only MBP with Wag31 and Wag31∆N, respectively. (**B**) The silver-stained SDS-PAGE depicts the precipitated protein elutes from the test and control reactions, set up as indicated in (**A**). The reaction contents are shown on top of each lane of the gel. The position of proteins is shown by arrow marks. Pure proteins are shown as indicated in the last four lanes.

Since N-terminal region containing the lipid binding domain of Wag31 is responsible for its localization in the cell membrane [35], a Wag31 deletant (Wag31∆N), lacking the N-terminal region was used to see whether the interaction between the two proteins was retained. The purification profile of Wag31∆N is shown in Figure S1. As the anti-Wag31 antibodies used for previous interactions showed very weak binding to Wag31∆N, we resorted to amylose beads-mediated affinity precipitation strategy. Fusion proteins comprising Rv3852 or Rv3852∆ctd and maltose-binding protein (Rv3852-MBP and Rv3852∆ctd-MBP) were generated for the purpose and used (Figure 3 and Figure S2). The reactions were set according to the schematic shown in Figure 3A. In the test sample A, Wag31 protein interacted with Rv3852∆ctd-MBP, while in test sample B, Wag31∆N did not interact with Rv3852∆ctd-MBP, suggesting that N-terminal region of Wag31 might contribute to the interaction of Wag31 with Rv3852 (Figure 3B). Similarly, Wag31∆N did not interact with Rv3852 (Figure S2). No interaction was seen with Wag31 and Wag31∆N in control reactions having only MBP protein (Figure 3B).

### 3.4. Rv3852 Influences Localization of Wag31 in the Mycobacterial Cell Wall

**Figure 4:**
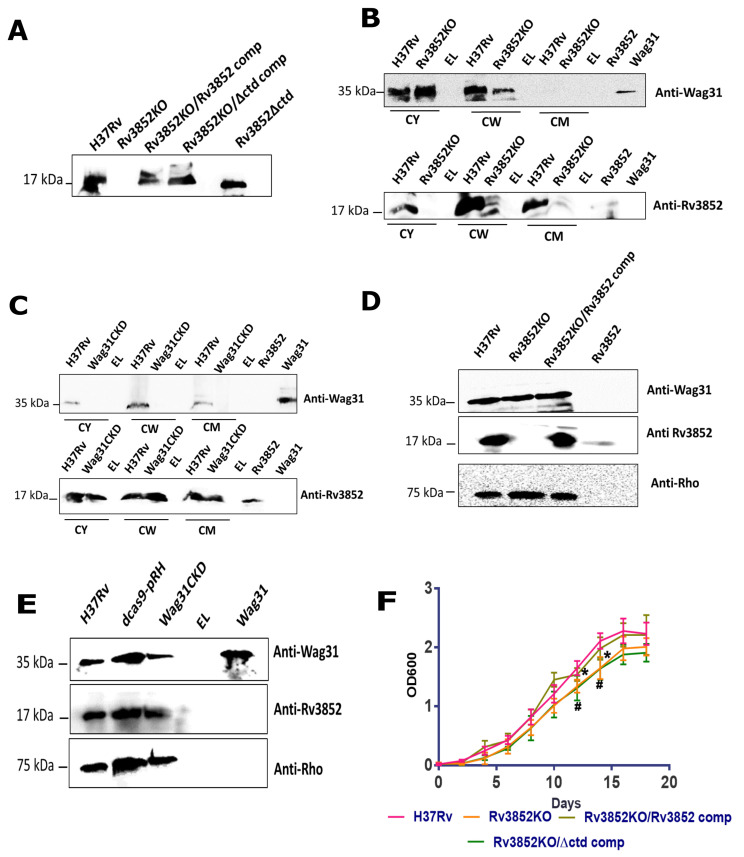
Rv3852 influences Wag31 localization in the Mtb cell wall. (**A**) The Western blot showing expression of Rv3852 and Rv3852∆ctd proteins in H37Rv, Rv3852KO, and the complemented strains. (**B**) The upper and lower immunoblots show distribution of Wag31 and Rv3852 in different cellular fractions of Mtb H37Rv and Rv3852KO, respectively. (**C**) The upper and lower immunoblots show distribution of Wag31 in different cellular compartments in Mtb H37Rv and Wag31CKD. In blots (B) and (C), CY, CW, and CM denote cytoplasmic, cell wall, and cell membrane fractions, respectively. EL stands for empty lane. (**D**) The immunoblot showing Wag31 levels in Rv3852KO as compared to Mtb H37Rv whole-cell lysates. (**E**) The immunoblot showing Rv3852 levels in Wag31CKD, as compared to Mtb H37Rv. Anti-Rho was used as loading control in (D) and (E). The lanes Rv3852 and Wag31 in (B–E) show pure proteins. The densitometry analysis for (B–E) is shown in Figure S3. (**F**) The growth curve of H37Rv, Rv3852KO, and the complemented strains. The experiments were carried out thrice, and mean OD values were plotted. Error bars show standard deviation. * and # show points of significant growth defect (*p* < 0.05) in Rv3852KO and Rv3852KO/∆ctd comp, respectively, as compared to H37Rv.

Earlier studies have proposed that Wag31 might act as a scaffold for positioning of partner proteins, leading to mycobacterial cell wall synthesis [27]. To understand the physiological role of interaction of Wag31 with Rv3852, we examined the sub-cellular localization of Wag31 in the knockout strain of Rv3852. Rv3852KO was complemented with Rv3852 (Rv3852KO/Rv3852comp) and Rv3852∆ctd (Rv3852KO/∆ctd comp). The level of each protein is restored up to the wild type (WT) levels in each of the complemented strains (Figure 4A). Previously, Wag31 has been detected in whole-cell lysates as well as membrane fractions in exponential phase Mtb H37Rv [39]. By sub-cellular fractionation, we found that Wag31 is distributed in the cytoplasmic, cell wall, and the cell membrane fractions in exponential phase Mtb H37Rv (Figure 4C and Figure S3B). In Rv3852KO, Wag31 was depleted in the cell wall fraction, indicating the role for Rv3852 in regulating Wag31 localization in the cell wall (Figure 4B and Figure S3A). To explore whether Wag31 impacts Rv3852 localization, we generated a conditional knockdown of Wag31 (Wag31CKD) using the CRISPR-Cas9 approach to deplete the levels of Wag31. The Western blots confirming Wag31CKD are shown in Figure S4. The amount of Rv3852 tethering to the cell wall did not change in Wag31CKD (Figure 4C and Figure S3B).

To confirm that the perturbation in sub-cellular localization of Wag31 in Rv3852KO was not due to Rv3852-mediated transcriptional regulation of *wag31* gene, we performed immunoblotting for these proteins in the whole-cell lysates of H37Rv, Rv3852KO, and Wag31CKD. There was no quantifiable change in the total levels of Wag31 in the lysates of Rv3852KO and Rv3852 in Wag31CKD lysate, indicating that the difference in concentration of Wag31 in cellular compartments is due to protein–protein interaction between them (Figure 4D,E and Figure S3C,D). Since Wag31 is an essential protein for mycobacterial survival, and its localization is disturbed in Rv3852KO, we assessed in vitro growth differences amongst various strains of Rv3852 as compared to WT. Both Rv3852KO as well as Rv3852KO/∆ctd comp showed small but consistent growth defect in late exponential phase as compared to H37Rv (Figure 4F).

### 3.5. Absence of Rv3852 Disturbs Polar Localization of Wag31

**Figure 5:**
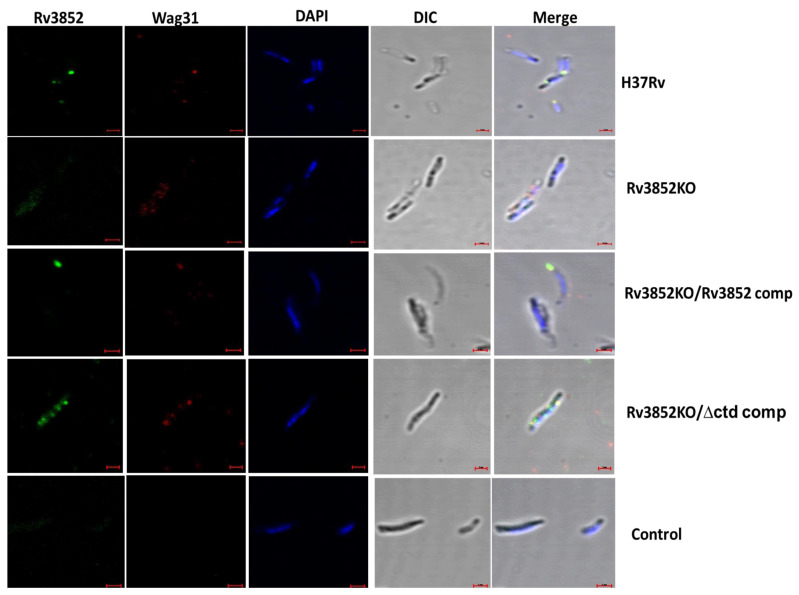
Mislocalization of Wag31 in absence of Rv3852 in Mtb. First and second panels show representative confocal images showing co-localization of Rv3852 (green) and Wag31 (red) at one or both poles of Mtb H37Rv and mislocalization of Wag31 in Rv3852KO, respectively. Third panel shows co-localization between Rv3852 and Wag31 in Rv3852KO/Rv3852 comp. Fourth panel shows diffused distribution of Rv3852∆ctd and Wag31. Pre-immune sera were used as negative control (last panel). The last lane of each panel shows merged image from all the channels. The images were acquired using a Zeiss LSM880 microscope under 100× objective with 2× zoom. Scale bar indicates 1 µm. See Figure S5 for fluorescence intensity distribution pattern.

Since the polar localization of Wag31 helps in organization of elongasome in mycobacteria [13,27], we examined the distribution of Rv3852 and Wag31 in the exponential phase Mtb H37Rv using immuno-fluorescence. Rv3852 formed discrete high-intensity foci at one or both the poles of the bacterium (Figure 5 and Figure S5A) and co-localized with Wag31 at poles of Mtb. Scatter plots of fluorescence intensity for Wag31 and Rv3852 along the length of mycobacteria (*n* = 28) showed overlapping distribution for both the proteins, mostly at the poles (Figure S5A). Given the decreased cell wall localization of Wag31 in Rv3852KO (results described in previous section) and co-localization of Rv3852 with Wag31, we next analyzed localization of Wag31 in absence of Rv3852. Wag31 was found to be localized away from the pole in Rv3852KO (*n* = 19; Figure 5 and Figure S5B). The polar localization of Wag31 was restored with the complementation of full-length Rv3852, as shown by the intensity scatter plots (*n* = 28; Figure 5 (third panel) and Figure S5C). Notably, in complementation experiments with the C-terminal deletant of Rv3852, instead of positioning in the poles, Wag31 followed the distribution profile of Rv3852∆ctd and formed multiple foci along the cell length (*n* = 25; Figure 5 (fourth panel) and Figure S5D).

### 3.6. Ectopic Expression of Rv3852 in M. smegmatis Leads to More DivIVA_Msm_ Deposition in Cell Wall

**Figure 6:**
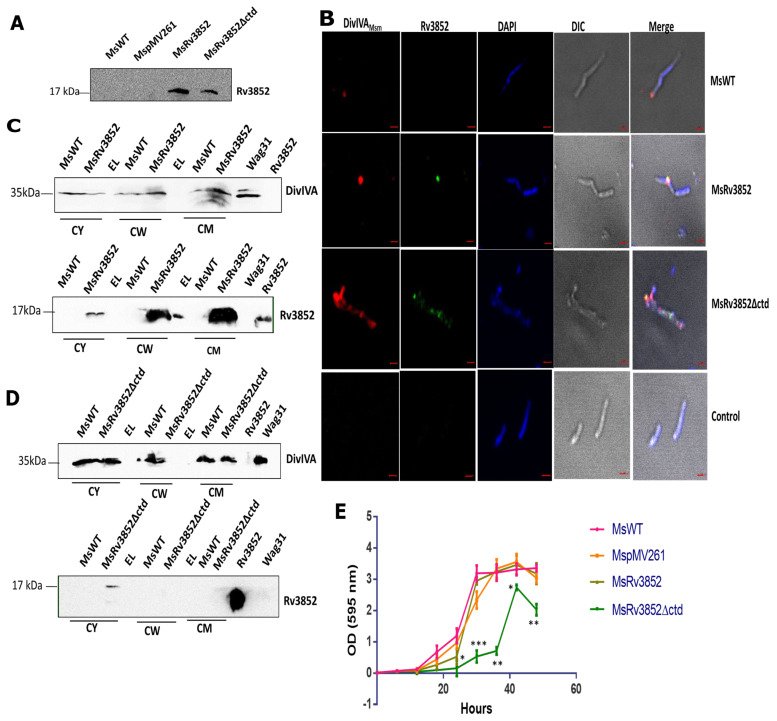
Ectopic expression of Rv3852 in *M. smegmatis* leads to more DivIVA_Msm_ deposition in cell wall. (**A**) Immunoblot showing the expression levels of Rv3852 and Rv3852∆ctd proteins in *M. smegmatis*. (**B**) Representative confocal images showing co-localization of Rv3852 (green) and DivIVA_Msm_ (red) in *M. smegmatis.* Top row: localization of DivIVA_Msm_. Second row: Co-localization between Rv3852 and DivIVA_Msm_ in MsRv3852. Third row: Co-localization between Rv3852∆ctd and DivIVA_Msm_ in MsRv3852∆ctd. Last row: Cells stained with pre-immune serum. The last vertical lane of each row shows merged image from all four channels. The images were acquired using a Zeiss LSM710 microscope under 100× objective with 2× zoom. Scale bar indicates 1 µm. See Figure S6 for fluorescence intensity distribution pattern for both the proteins in all the three strains. (**C**) Immunoblot showing increased localization of DivIVA_Msm_ in the membrane and the cell wall fractions of MsRv3852 as compared to *M. smegmatis*. The cytoplasmic (CY), cell wall (CW), and cell membrane (CM) fractions are indicated in (C) and (D). EL stands for empty lane in (C) and (D). (**D**) Immunoblot showing localization of DivIVA_Msm_ in different cellular compartments in MsRv3852∆ctd strain. The densitometry analysis for (C) and (D) is shown in Figure S7. Anti-Wag31 antibody was used for detecting DivIVA_Msm_. Rv3852 and Wag31 lanes in (C), (D), and (E) show respective pure proteins. (**E**) The growth curve of different *M. smegmatis* Rv3852 strains. The experiment was performed thrice, and mean OD values were plotted. Error bars show standard deviation. *, **, and *** indicate OD with *p* values <0.05, <0.01, and <0.001, respectively, in MsRv3852∆ctd as compared to MsWT.

In our immuno-precipitation experiments using MsRv3852, we could retrieve DivIVA_Msm_ in the pellet fraction (Figure 1B). Taking this observation further, localization of Rv3852 was analyzed in MsRv3852 and MsRv3852∆ctd strains (ectopically expressing Rv3852 and Rv3852∆ctd proteins). The expression was confirmed by immunoblotting using anti-Rv3852 antibodies (Figure 6A). Rv3852 co-localized with DivIVA_Msm_, forming high-intensity foci at the cell poles in MsRv3852 strain (Figure 6B), akin to co-localization with Wag31 in Mtb. The fluorescence intensity plot for both the proteins is shown in Figure S6B (*n* = 22). DivIVA_Msm_ was more diffused and co-localized with Rv3852∆ctd on the nucleoid (*n* = 10, Figure 6B and Figure S6C). The expression of Rv3852 led to increased cell wall tethering of DivIVA_Msm,_ (Figure 6C and Figure S7A), while in MsRv3852∆ctd strain, DivIVA_Msm_ was found to be predominantly localized to the cytoplasm, possibly because the cognate partner Rv3852∆ctd is present in the cytoplasm (Figure 6D and Figure S7B). To assess the effect of perturbation of localization of DivIVA_Msm_ on *M. smegmatis* growth, we compared the growth pattern of the strains. The MsRv3852 strain showed comparable growth profile with MspMV261 (only vector) strain. However, the MsRv3852∆ctd showed severe growth defect, likely due to sequestration of DivIVA_Msm_ from its site of action (Figure 6E) (see the Discussion section).

### 3.7. Absence of Rv3852 Affects Nascent Peptidoglycan Synthesis

**Figure 7:**
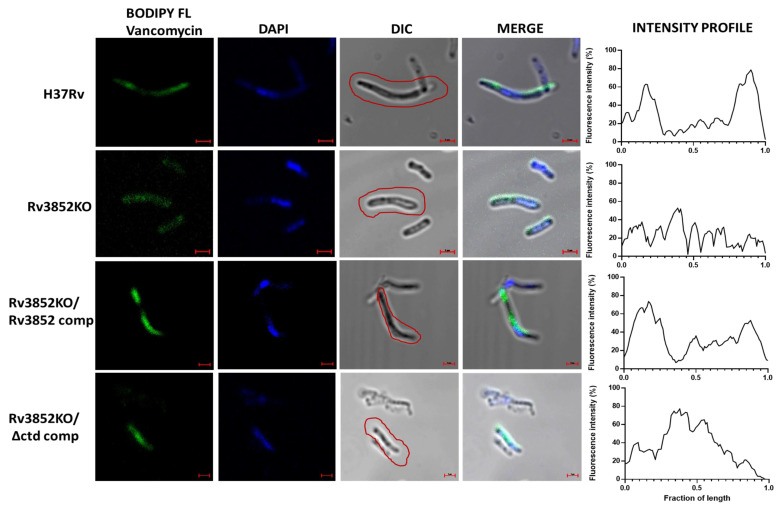
Absence of Rv3852 affected nascent peptidoglycan synthesis. Representative confocal images of BODIPY FL Vancomycin staining of Mtb H37Rv, Rv3852KO, Rv3852KO/Rv3852 comp, and Rv3852KO/∆ctd comp strains. Red boundaries indicate the cells taken for intensity profile measurement shown in the last lane of each panel. Bacterial length is normalized to 1, and the fluorescence intensity is shown as percentage of highest intensity. The curve was smoothed using Graph Pad Prism 6.0. The merge lane shows merged image of all channels. The images were acquired using a Zeiss LSM880 microscope under 100× objective with 2× zoom. Scale bar indicates 1 µm. See Figure S8 for fluorescence intensity distribution pattern.

Although Wag31 does not have any known enzymatic activity, its depletion is manifested as a defect in polar peptidoglycan synthesis [13,27]. Taking a cue from this, we analyzed whether perturbation in Wag31 localization leads to any changes in nascent peptidoglycan synthesis. For this, exponential phase cells of Mtb and *M. smegmatis* strains containing various Rv3852 constructs were grown in the presence of BODIPY FL Vancomycin, which fluorescently labels extracellular, lipid-linked peptidoglycan precursors [37,40]. Mtb H37Rv showed staining on the poles and along the walls (Figure 7 and Figure S8A), while Rv3852KO showed diffused BODIPY FL staining, indicating disturbed cell wall synthesis (Figure 7 and Figure S8B). Complementation with Rv3852 restored the polar and the lateral BODIPY FL signal (Figure 7 and Figure S8C), while complementation with Rv3852∆ctd showed signal away from the poles (Figure 7 and Figure S8D), owing to the co-localization of Wag31 with Rv3852∆ctd (see Figure 5). MsRv3852 strain showed stronger BODIPY FL staining, likely due to more cell wall tethering of both Rv3852 and DivIVA_Msm_. In contrast, MsRv3852∆ctd strain showed disordered nascent peptidoglycan staining, possibly due to perturbation in DivIVA_Msm_ localization (Figure S9). Thus, ectopic expression in *M. smegmatis* of the two alleles of Rv3852, especially the deletant (Rv3852∆ctd), provides insight into the importance of the interaction between the two partner proteins. In MsRv3852∆ctd strain, bulging at the cell poles was seen most likely due to mis-localization of DivIVA_Msm_ away from the poles (Figure S9, MsRv3852∆ctd panel). In contrast, we did not observe change in cell shape in MsRv3852, as in this case, full-length NAP interacted with DivIVA_Msm_ at poles without sequestering it away from its site of location (see the Discussion section). From all these results, it is apparent that Rv3852 possibly acts as a driver of Wag31 to cell poles, where the latter is known to interact with cell wall biosynthetic machinery.

## 4. Discussion

We describe an unusual interaction between a NAP and a cell pole-localized protein needed for cell elongation in Mtb. This specific interaction appears to be regulating Wag31 localization and its function in cell wall biogenesis. While Wag31 is a well-studied protein with established functions in mycobacteria and in other bacterial species, Rv3852 is a DNA-binding protein with limited information and is confined to a few species of mycobacteria. However, unlike other NAPs, with the aid of its C-terminal stretch, it anchors itself to the cell wall while it is bound to DNA [10]. This unusual localization of Rv3852 suggests a new role for it other than DNA binding and chromosome organization properties. While Wag31 is an essential cell division protein that coordinates polar growth in Mtb [13,35], and is the key player in the lateral cell wall biogenesis at the cell poles to elongate the growing Mtb cells [27], the NAP appears to not be essential for bacterial survival.

The impact of knocking out Rv3852 in Mtb is manifested in the form of perturbed polar localization of Wag31 (Figure 5). Rv3852KO showed small but consistent growth defect (Figure 4F). However, the complementation of the knockout with full-length Rv3852 restored the growth defect, but the truncated version complementation continued to show growth defect, most likely due to over-condensation of the nucleoid and co-localization of Wag31 and Rv3852∆ctd over the nucleoid (Figure 5). Thus, Rv3852∆ctd serves to selectively deplete Wag31 at its site of action, leading to inadequate localization at the cell pole. This possibly leads to inadequate and inefficient positioning of partner proteins, leading to the observed defect.

Although found only in slow growing pathogenic mycobacteria and not in *M. smegmatis* and other fast-growing species of the genus, when expressed in *M. smegmatis*, Rv3852 interacted with DivIVA_Msm_, an orthologue of Wag31. Thus, ectopic expression of Rv3852 in *M. smegmatis* is insightful in assessing the significance of the interaction between the NAP and Wag31. As in the case of Mtb, Rv3852 co-localized with DivIVA_Msm_ at the poles of the *M. smegmatis* (Figure 6). Moreover, with this co-localization, it ensured increased tethering of DivIVA_Msm_ to the cell wall (Figure 6). The expression of Rv3852 in *M. smegmatis* did not perturb the bacterial growth, while the MsRv3852∆ctd showed substantial growth defect and bulging phenotype, most likely due to sequestration of DivIVA by the truncated NAP away from its normal site. Such bulged cells due to altered cell shape is not seen with Rv3852KO, its complemented strains, or when it is ectopically expressed in *M. smegmatis,* because in these cases Wag31 localization is not impacted severely. This perturbation seen in DivIVA localization is suggestive that such an interaction is not warranted in *M. smegmatis* and that Wag31 regulation by the NAP may be confined to Mtb. While it is conceivable that *M. smegmatis* may have evolved alternate modes to regulate polar regulation of DivIVA, given the differences in their rate of cell division, growth, and other metabolic activities, the Wag31–Rv3852 nexus appears to be exclusive to Mtb. Although much of the cell division components and cell wall biosynthesis machinery are conserved between the two species, the process itself may need to be regulated differently between the two well-studied species of mycobacteria. Thus, although Wag31 and DivIVA are engaged in similar function in Mtb and *M. smegmatis*, respectively, in maintaining cell pole integrity, the interaction of Rv3852 with Wag31 in Mtb adds another puzzle to the diversity of Wag31 function.

As Wag31/DivIVA interact with a range of proteins involved in exclusive polar growth and maintenance of cellular morphology [13,41,42,43,44,45]; cell wall synthesis; and cell division such as AccA3 [46], CwsA [47], and chromosomal partition protein ParA [30], their localization at the poles may be of crucial importance. CwsA (Rv0008c/MSMEG_0023) along with CrgA function in localizing Wag31 to its action site [48]. Deletions of CwsA and/or CrgA resulted in defects in polar localization of Wag31 and cell wall synthesis [48]. By interacting with Wag 31, CwsA directly promotes Wag31 localization, while the role of CrgA appears to be indirect. Thus, in addition to Rv3852, other components in mycobacteria are involved in correct positioning of Wag31. Hence, Rv3852KO had only minor growth defect and some effect on Wag31 localization. However, with the C-terminal-truncated mutant of Rv3852, more severe growth defect as well as altered distribution of Wag31 is seen. Thus, the interaction of Rv3852 with Wag31 basically adds another layer to this complex network. Rv3852 participation in this network of polar growth and cell division in Mtb by interacting with Wag31 could have synergistic or antagonistic consequences. These tantalizing possibilities would be a subject of future study. Importantly, the Rv3852 mutants described here would serve as invaluable tools in understanding its cellular function and as probes to explore the diverse roles exhibited by Wag31/DivIVA_Msm_ in the two species of mycobacteria.

Apart from being a chromatin organizer, few of the NAPs appear to have taken up additional functions in different species of bacteria. In *Caulobacter crescentus*, an AT-rich sequence-binding transcription regulator, GapR, participates in cell cycle progression [49]. GapR is also considered to be a NAP and is a master regulator for switching over to swarmer cells from stalk cells, accumulating preferentially in pre-divisional cells [49]. GapR also binds to *Caulobacter* origin of replication (*Cori*) and separates newly duplicated chromosomes before parABS-mediated partitioning sets, as shown in [50]. In *E.coli*, apart from its DNA-bending function, Fis, is also involved in phage lambda site-specific recombination, transcriptional activation of rRNA and tRNA operons, and OriC-directed DNA replication [51]. DivIVA also interacts with a few other DNA-binding proteins in different bacteria. RacA from *B. subtilis* is one of them [21,22,52]. RacA is expressed during sporulation of *B. subtilis* and binds to specific DNA sequences termed RacA-binding motifs or rams. Interaction of RacA with DivIVA helps in its membrane tethering, leading to anchorage of divided DNA at the two poles [21,22,52]. However, RacA is not a NAP, but it also tethers to membrane. Thus, it seems to carry out an analogous function of Rv3852 in *B. subtilis*.

Finally, the druggability of Wag31 was demonstrated elegantly by phenotypic screening of a compound library against replicating Mtb [35]. Aminopyrimidine sulfonamides showed potent bactericidal activity against Mtb, and the resistant mutations mapped exclusively to the *wag31* gene. Moreover, the inhibitors disturbed the old poles of Mtb cells. However, after an in-depth analysis, Singh et al. concluded that Wag 31 is not the direct target for aminopyrimidine sulfonamides and suggested several plausible mechanisms of action for the inhibitors [35]. They also suggested that these molecules may impair protein–protein interactions or inhibit by a yet unknown mechanism. It remains to be seen whether these small molecule inhibitors impact the interaction between Rv3852 and Wag31. Nevertheless, it appears that targeting Wag31–Rv3852 interaction or the individual proteins would be a step forward in efforts to find new ways of affecting Mtb growth.

## Data Availability

The data presented in this study are available in this article and Supplementary Materials only.

## Supplementary Materials

The following are available online at https://www.mdpi.com/article/10.3390/cells10061558/s1, Figure S1: Purification of proteins, Figure S2: Rv3852 interacts with N-terminal region of Wag31, Figure S3: Densitometry analysis for proteins in western blots in Figure 4, Figure S4: Generation of Wag31CKD in Mtb H37Rv, Figure S5: Scatter plot for fluorescence intensities of Rv3852 and Wag31 along cell length in Mtb Rv3852 strains in Figure 5, Figure S6: Scatter plot of fluorescence intensities of Rv3852 and Wag31 along cell length in *M. smegmatis* strains in Figure 6, Figure S7: Densitometry analysis for proteins in western blots in Figure 6, Figure S8: Fluorescence Intensity plots of BODIPY FL Vancomycin staining of Mtb strains in Figure 7, Figure S9: Nascent peptidoglycan staining of different *M. smegmatis* strains, Table S1: List of oligonucleotides used in the study.
