# Supplementary material for: Direct Interaction of Polar Scaffolding Protein Wag31 with Nucleoid-Associated Protein Rv3852 Regulates Its Polar Localization
Source: Cells. 2021 Jun 20;10(6):1558. doi: 10.3390/cells10061558 (PMC8233713; doi:10.3390/cells10061558)
Supplement: Supplementary file 1 [file cells-10-01558-s001.zip › cells-1251595-supplementary.pdf]

Supplementary material

Direct interaction of polar scaffolding protein Wag31 with Nucleoid Associated Protein Rv3852 regulates its polar localization

Rajni Garg [1,2], Chinmay Anand [1], Sohini Ganguly [1], Sandhya Rao [1], Rinkee Verma [1] and Valakunja Nagaraja [1,2,*]

[1]Department of Microbiology and Cell biology, Indian Institute of Science, Bengaluru, Karnataka, 560012, India
[2]Jawaharlal Nehru Centre for Advanced Scientific Research, Bengaluru, Karnataka, 560064, India
*Correspondence: vraj@iisc.ac.in; Tel.: +91 80 23600668; Fax: +91 80 23602697

Supplementary materials: Fig S1-S9; Table S1

Fig. S1 Purification of proteins
Fig. S2 Rv3852 interacts with N-terminal region of Wag31
Fig. S3 Densitometry analysis for proteins in western blots in Figure 4
Fig. S4 Generation of Wag31CKD in Mtb H37Rv
Fig. S5 Scatter plot for fluorescence intensities of Rv3852 and Wag31 along cell length in Mtb Rv3852 strains in Figure 5
Fig. S6 Scatter plot of fluorescence intensities of Rv3852 and Wag31 along cell length in *M. smegmatis* strains in Figure 6
Fig S7. Densitometry analysis for proteins in western blots in Figure 6
Fig S8: Fluorescence Intensity plots of BODIPY FL Vancomycin staining of Mtb strains in Figure 7
Fig S9. Nascent peptidoglycan staining of different *M. smegmatis* strains
Table S1: List of oligonucleotides used in the study

Fig. S1 Purification of proteins

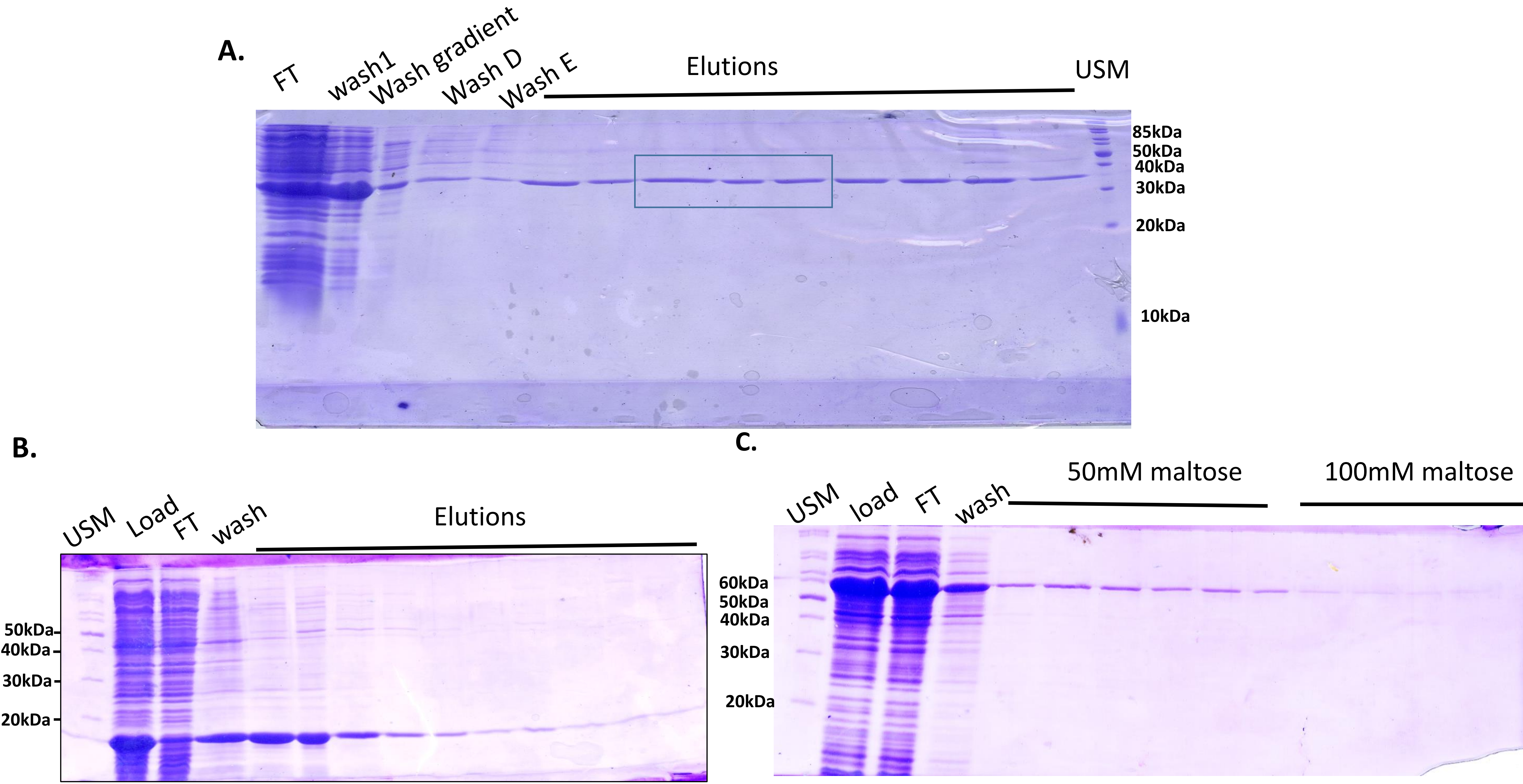

Fig. S1. Purification of proteins. (A) Coomassie brilliant blue stained SDS-PAG showing elution profile of Wag31. BL21 Cells were induced with 0.5 mM IPTG and purification was carried out as described in Materials and Methods. Protein was denatured using 8 M urea, followed by on-column renaturation. (B) Coomassie brilliant blue stained SDS-PAG showing the elution profile of Wag31ΔN. (C) Elution profile of Rv3852ΔctdMBP. Protein was eluted using 50 mM and 100 mM maltose.

Fig. S2 Rv3852 interacts with N-terminal region of Wag31

A.

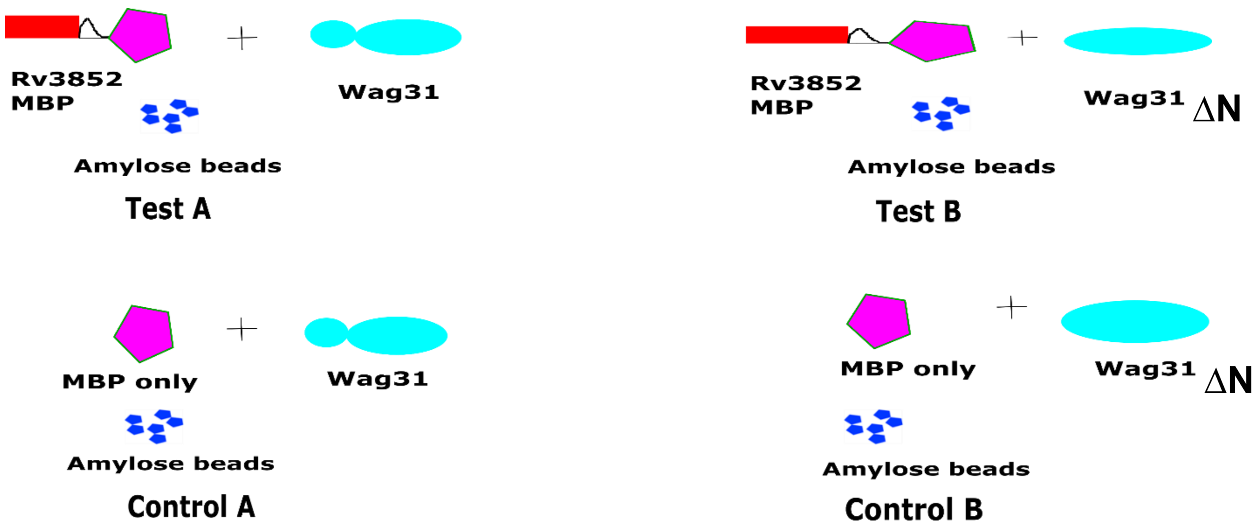

B.

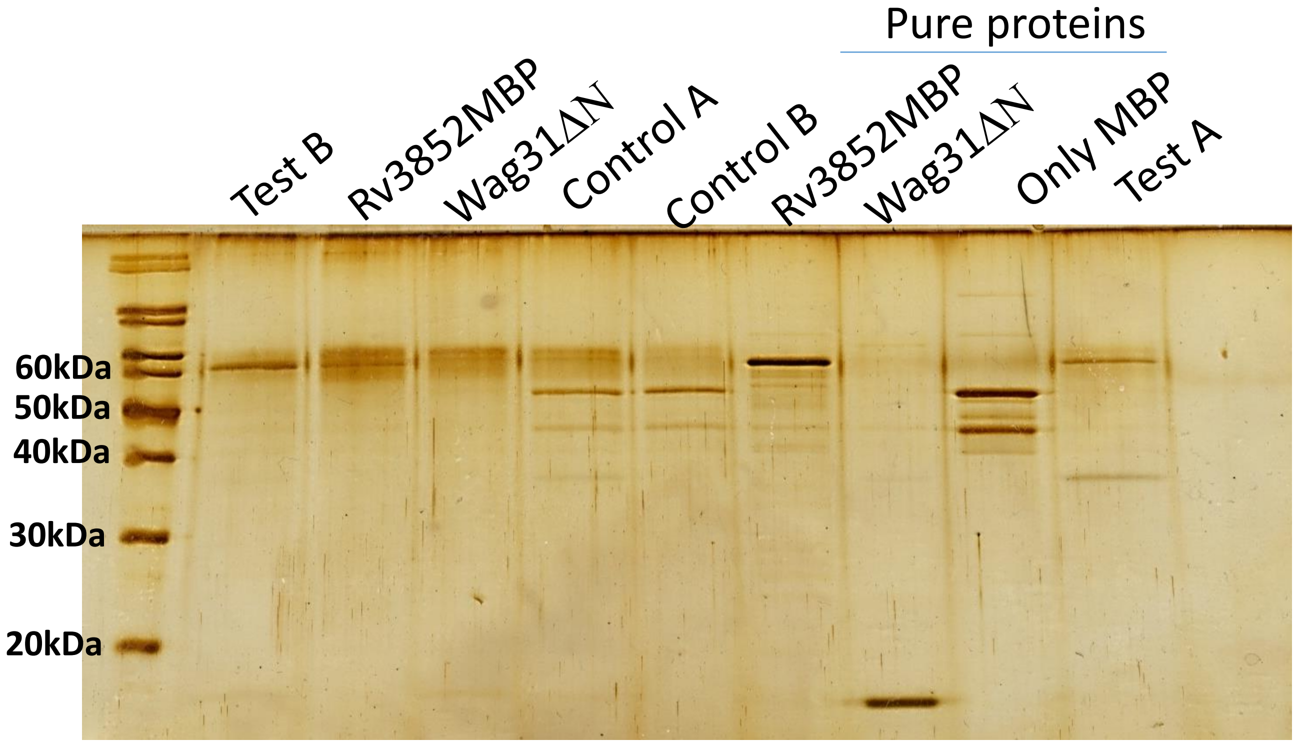

Fig. S2. Rv3852 interacts with N-terminal region of Wag31. The co-immunoprecipitation reactions using amylose beads were carried out as indicated in the schematic A. The test reactions A and B contained Rv3852-MBP with Wag31 and Rv3852-MBP with Wag31ΔN respectively. The control pairs A and B show negative control reactions having only MBP with Wag31 and Wag31ΔN. (B) The silver-stained SDS-PAG show the precipitated protein elutes from the test and control reactions set up as indicated in the diagram A. All the reactions contained amylose beads and purified proteins. The pure protein lanes contain indicated purified proteins.

Fig. S3 Densitometry analysis for proteins in western blots in Figure 4

A. Wag31 levels in Rv3852KO Fig. 4B

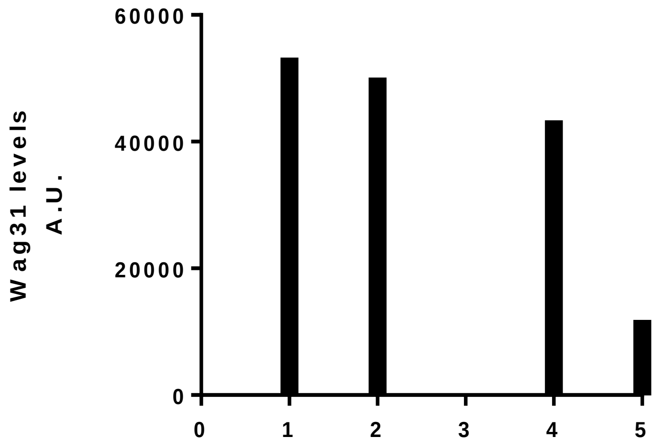

B. Rv3852 levels in Wag31CKD in Fig. 4C

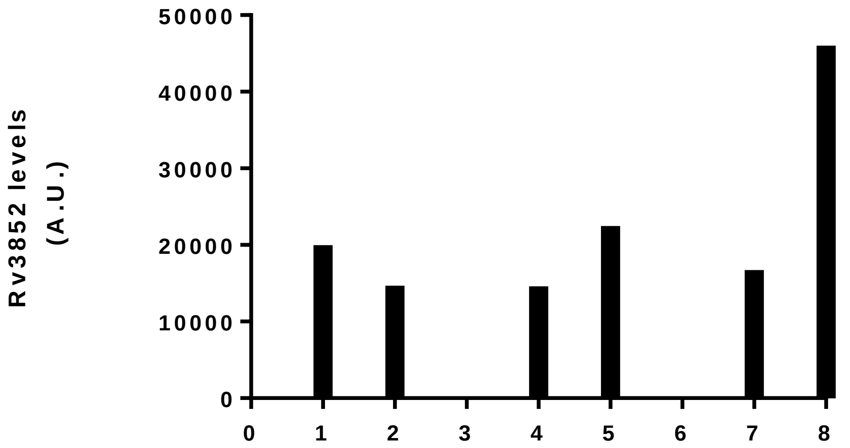

C. Wag31 levels in whole cell lysate in Fig. 4D

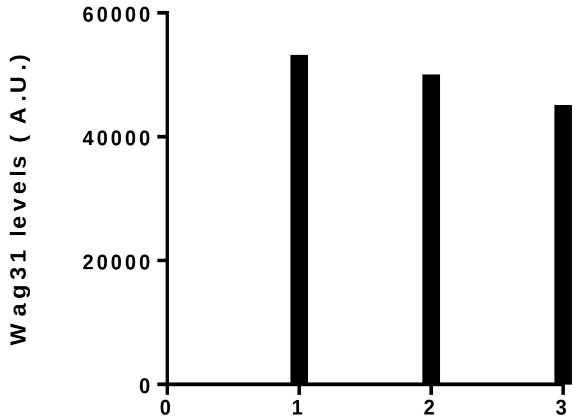

D. Rv3852 levels in whole cell lysate in Fig. 4E

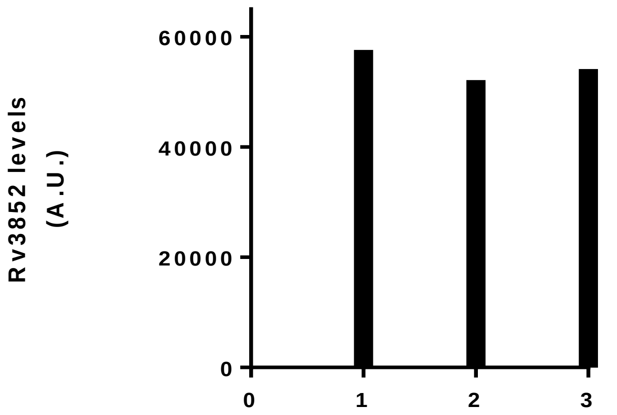

Densitometry analysis for proteins in western blots in Figure 4. The histograms for densitometry plots for western blots in Fig. 4B, 4C, 4D and 4E. The blots were quantified using ImageJ software. The numbers on X-axis indicate the lane numbers in the respective western blots.

Fig. S4 Generation of Wag31CKD in Mtb H37Rv

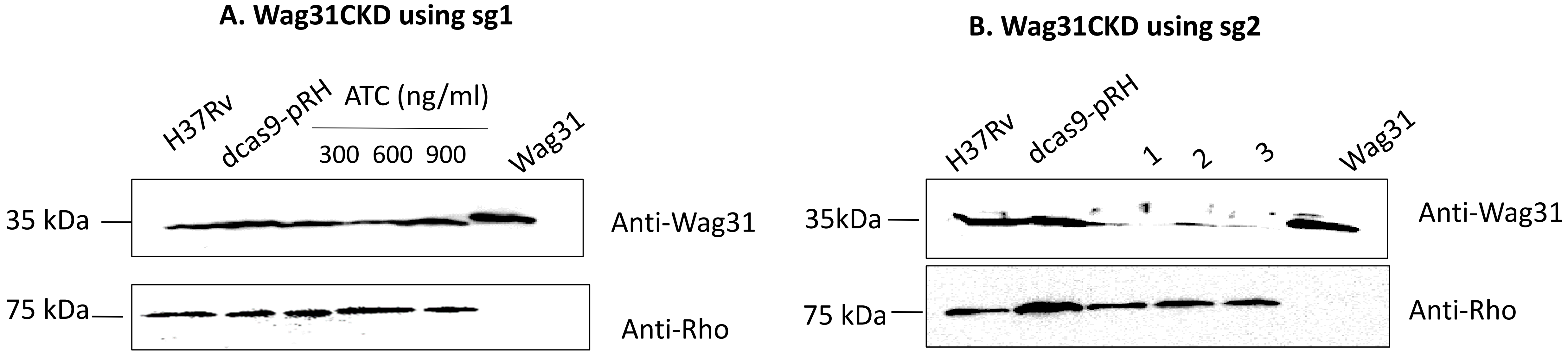

Fig S4. Generation of Wag31 conditional knockdown (Wag31CKD) in Mtb H37Rv. Western blot showing decrease in Wag31 levels in the cell lysates using guide RNAs: sg1 **(A)** and sg2 **(B)**. ATc concentrations are indicated in A. 600 ng/ml ATc was used in B. Pure Wag31 is included in the last lanes of both the blots. Rho was used as a loading control.

Fig. S5 Scatter plot for fluorescence intensities of Rv3852 and Wag31 along cell length in Mtb Rv3852 strains in Figure 5

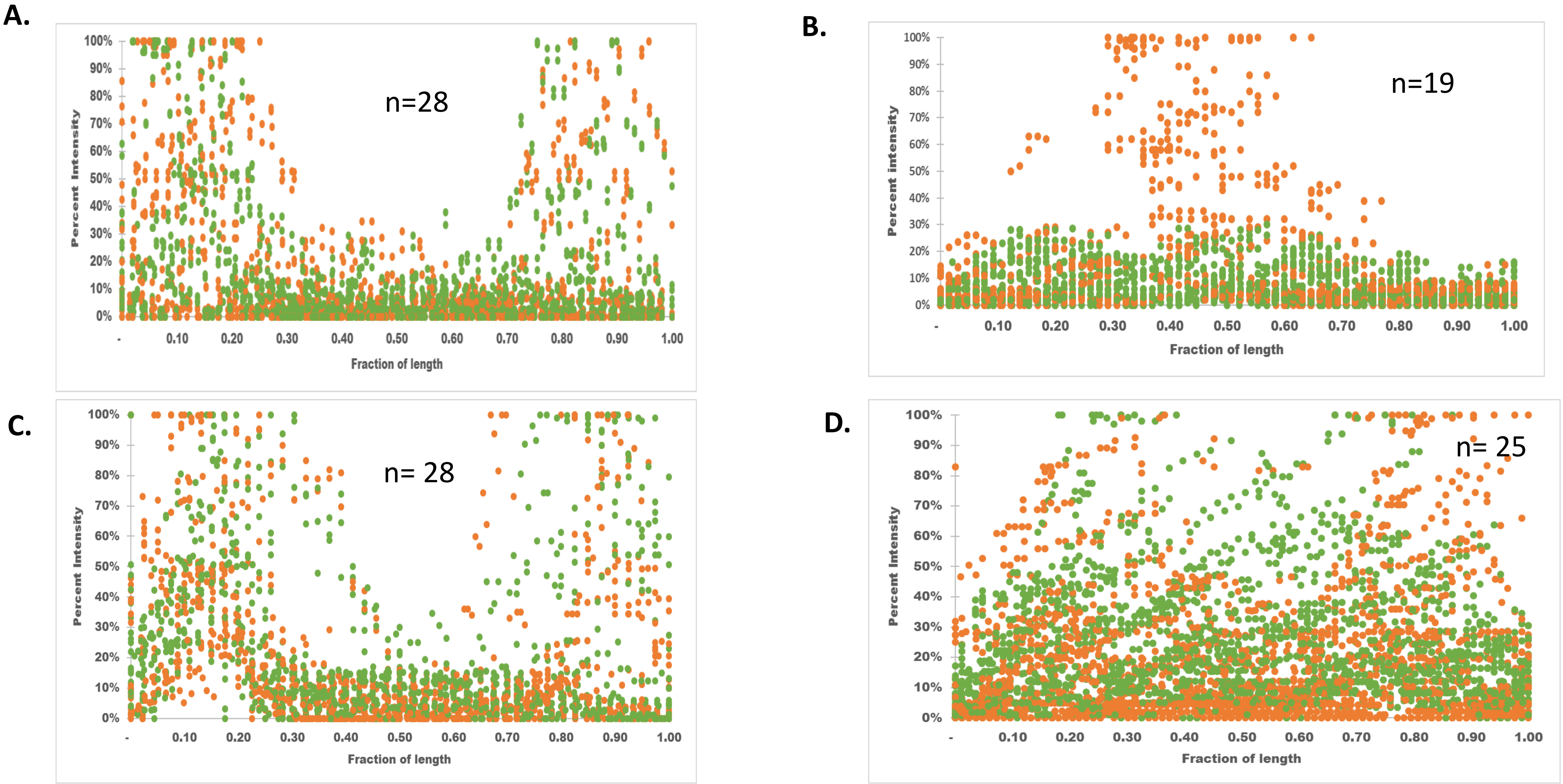

Fig. S5. Scatter plot for immunofluorescence intensities for Rv3852 and Wag31 along the cell length of Mtb H37Rv (A), Rv3852KO (B), Rv3852KO/Rv3852 comp (C) and Rv3852KO/Δctd comp strains (D) in main Figure 5. Each dot (orange for Wag31 and green for Rv3852) represents the fluorescence intensity expressed in % of the highest intensity values for respective proteins along the long axis of bacterium. Each dot was plotted against its position in the bacterium with the length of bacteria normalized to 1.

Fig. S6 Scatter plot of fluorescence intensities of Rv3852 and Wag31 along cell length in *M. smegmatis* strains in Figure 6

A.

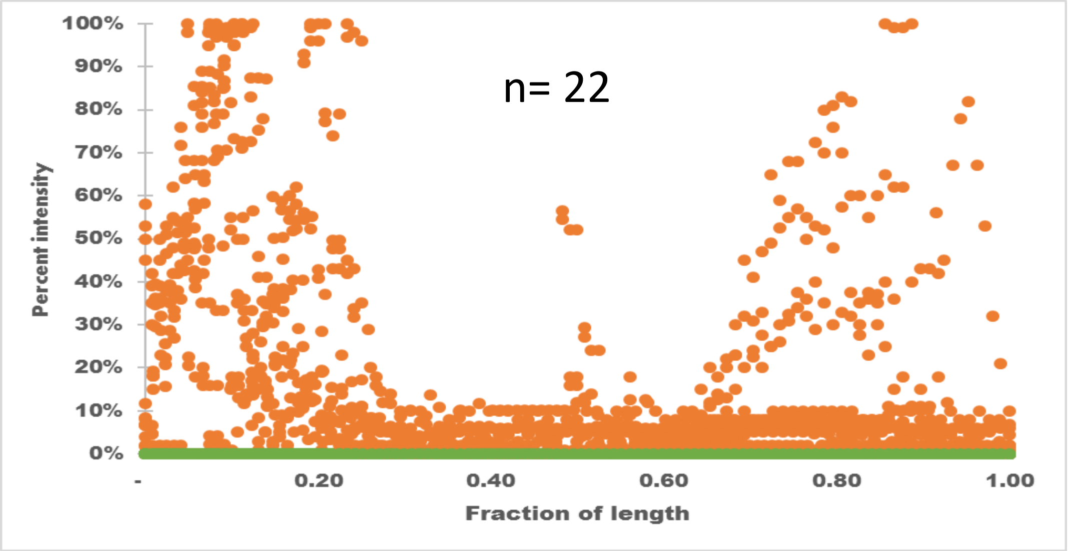

B.

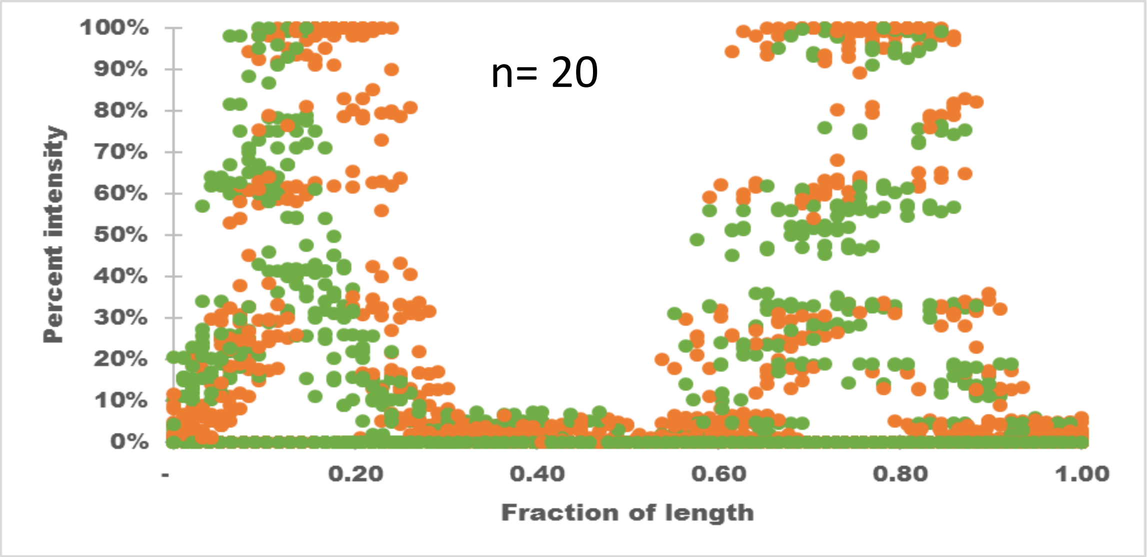

C.

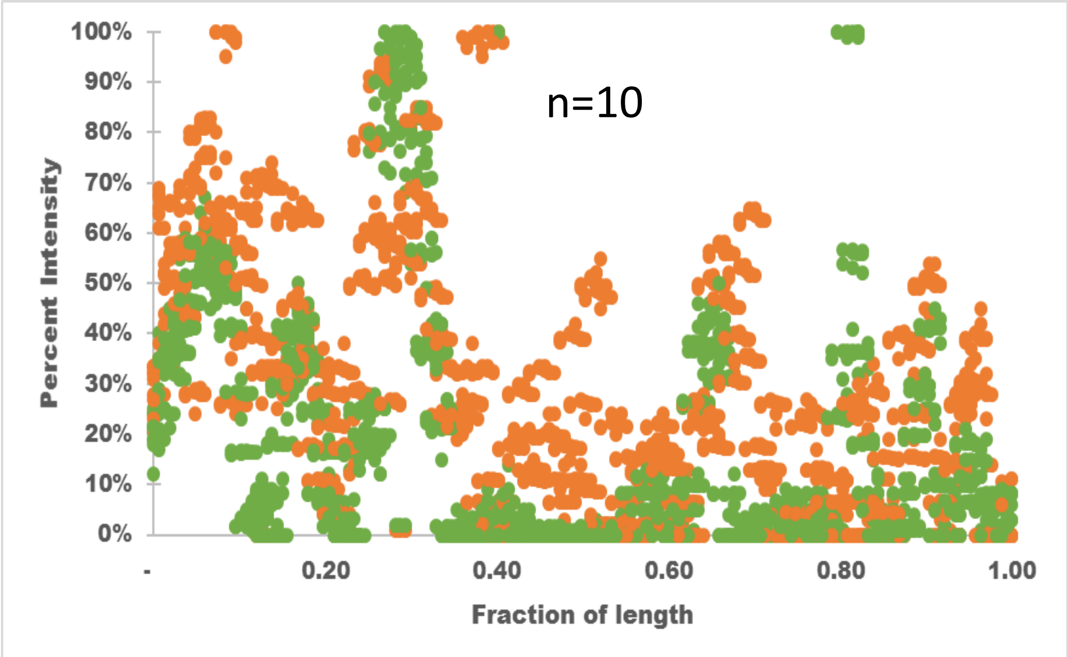

Fig. S6. Scatter plot of immunofluorescence intensities of Rv3852 and Wag31 along the cell length of *M. smegmatis* WT (A), MsRv3852 (B), MsRv3852Δctd strains (C), in main Figure 6. Each dot (orange for Wag31 and green for Rv3852) represents the fluorescence intensities expressed in % of the highest intensity values for respective proteins along the long axis of bacterium as described in Fig. S5.

Fig. S7 Densitometry analysis for proteins in western blots in Figure 6

A. DivIVA levels in MsRv3852 strain in Fig. 6C

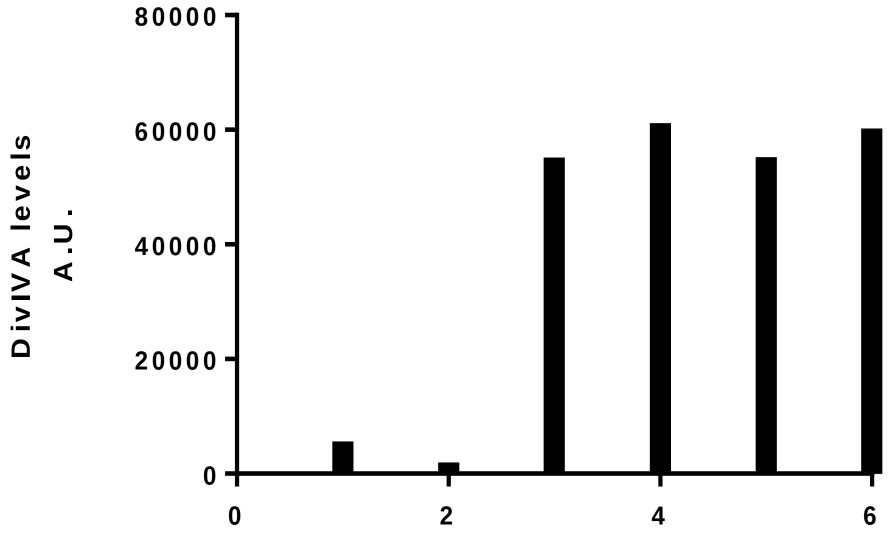

B. DivIVA levels in MsRv3852Δctd strain in Fig. 6D

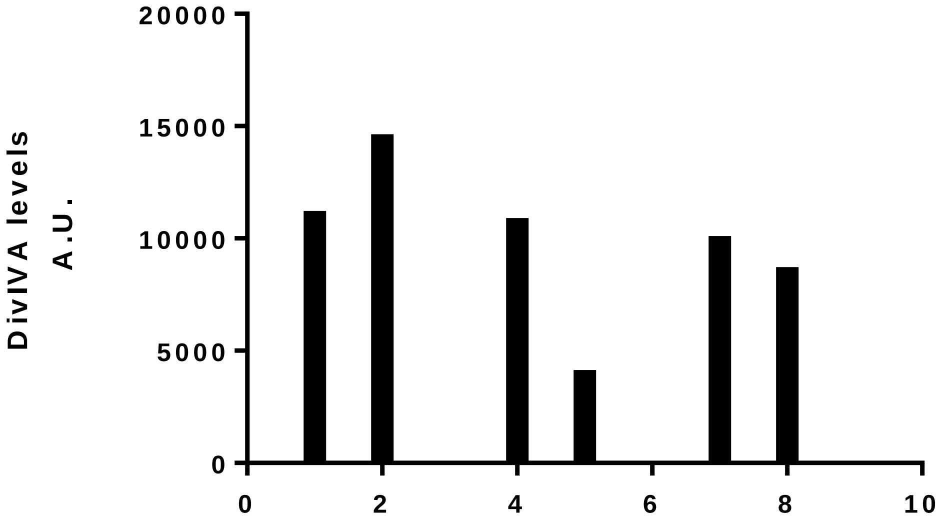

Fig S7. Densitometry analysis for proteins in western blots in Figure 6. The densitometry plots of western blots in Fig. 6C and 6D. The blots were quantified using ImageJ software. The numbers on X-axis indicate the lane numbers in the respective western blots.

Fig S8: Fluorescence Intensity plots of BODIPY FL Vancomycin staining of Mtb strains in Figure 7

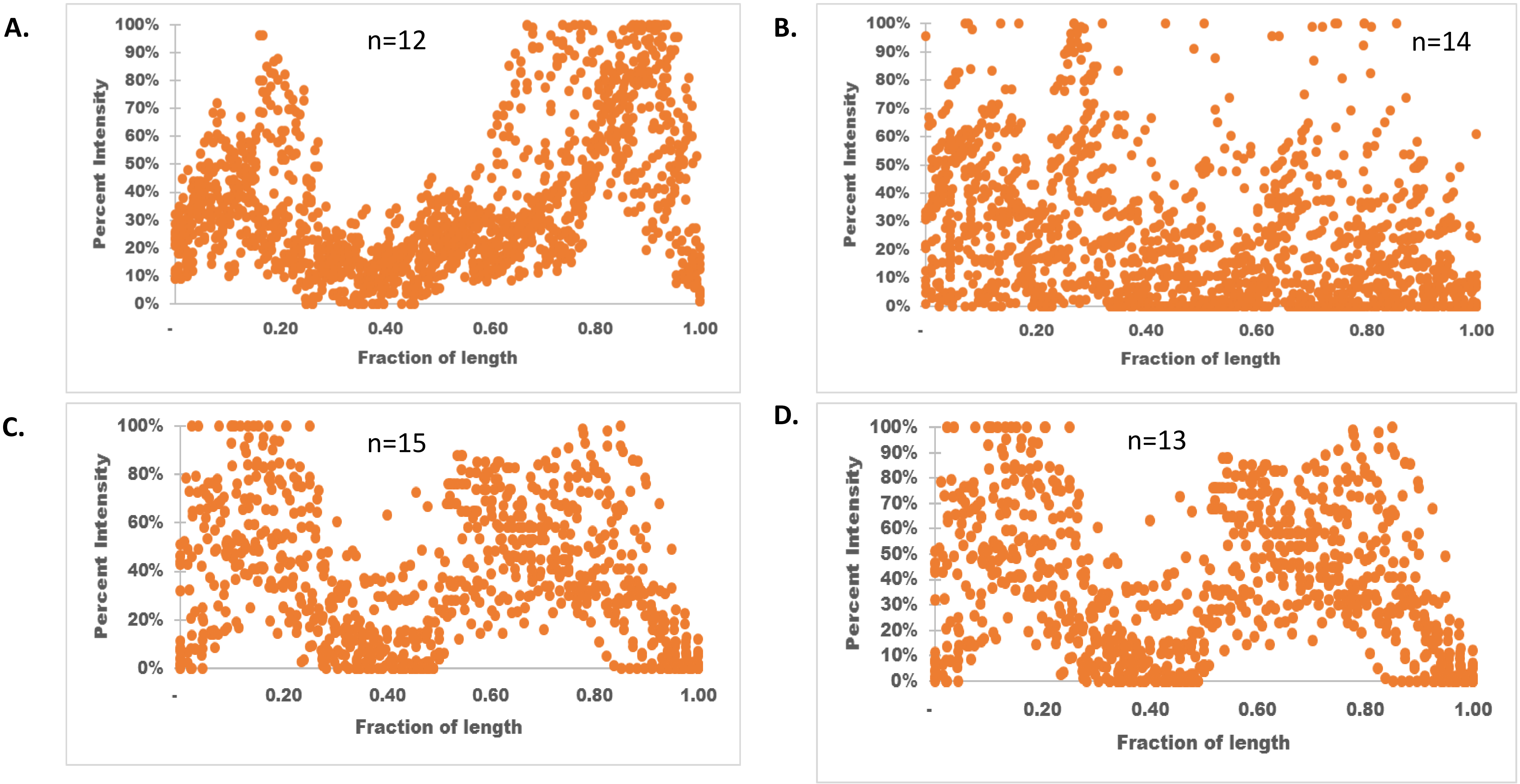

Fig. S8 Fluorescence Intensity plots for BODIPY FL Vancomycin staining along the cell length of Mtb H37Rv (A), Rv3852KO (B), Rv3852KO/Rv3852 comp (C), Rv3852KO/ Δctd comp strains (D), expressed in % of the highest values in main Figure 7. Each dot represents the BODIPY FL fluorescence intensity expressed in % of the highest intensity values along the long axis of bacterium. Each dot was plotted against its position in the bacterium with the length of bacteria normalized to 1.

Fig S9. Nascent peptidoglycan staining of different *M. smegmatis* strains

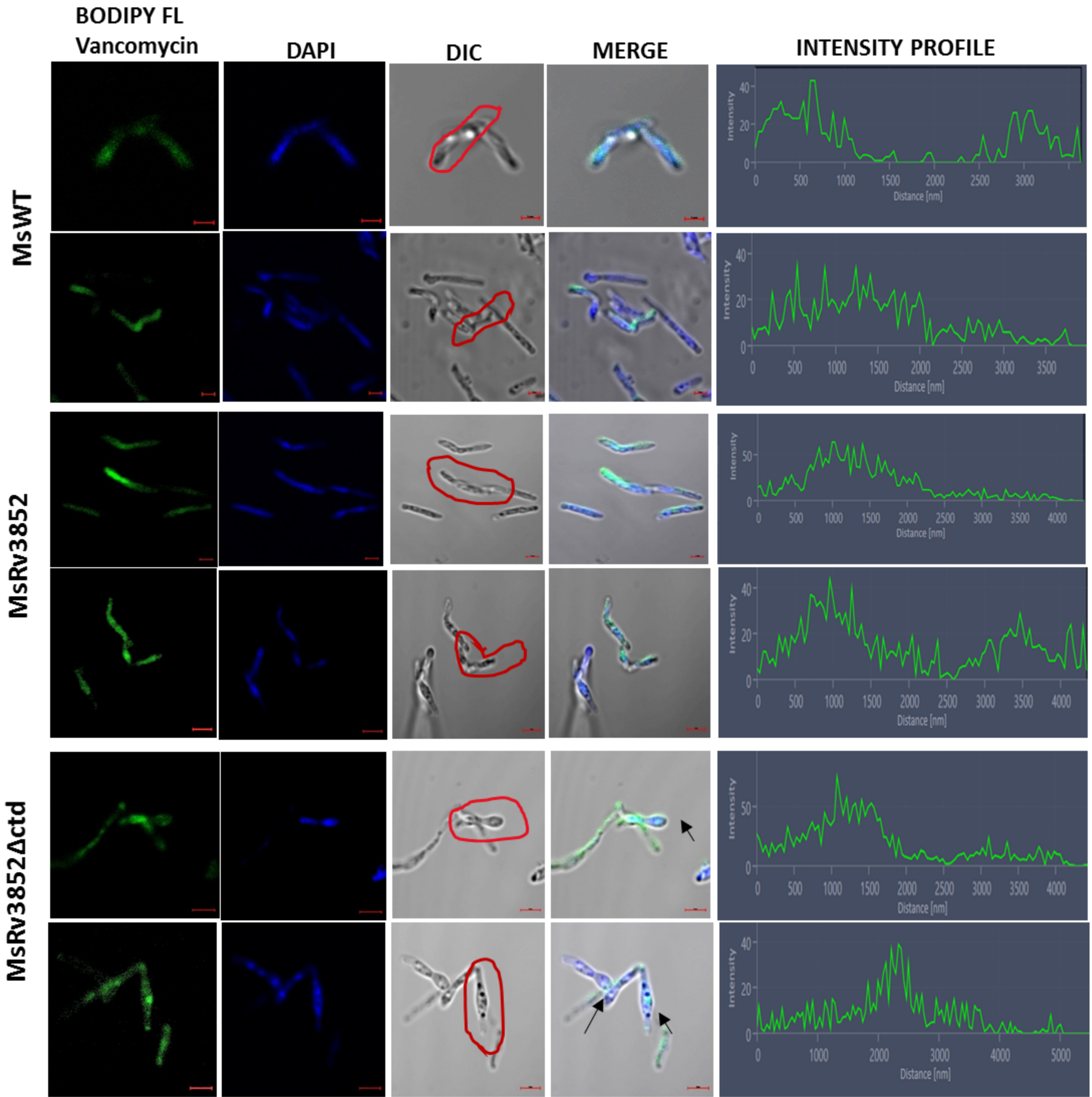

Fig S9. **Nascent peptidoglycan staining of different *M. smegmatis* strains**. MsWT, MsRv3852 and MsRv3852Δctd strains were grown till early log phase followed by addition of BODIPY FL Vancomycin for 3 h. The cells were fixed with 4% PFA and stained with 10 µg ml^{-1} DAPI for 30 min. Images were taken under 100X objective with 2X zoom using Zeiss LSM-880 Confocal microscope. The fluorescence intensity plot along the long axis of the bacterium (shown by red boundary) is shown in the last lane of all panels. Scale bars indicate 1µm. Only representative panels (two each) for three strains- MsWT, MsRv3852, MsRv3852Δctd are shown. Bulged cells are shown by black arrow heads.

Table S1: List of oligonucleotides used in the study

	Oligo name	Sequence
1	3852 pmv261 FP	5’GGCCGGATCCAATGCCAGACCCGCAGGAT 3’
2	3852 pmv261 RP	5’ GGCCAAGCTTTCAGCGGCGGCGCAGTTG 3’
3	3852 dctd pmv261 RP	5’ GGCCAAGCTTTCACGTGACGGCAACGAT 3’
4	Rv3852 MBP FP	5’GGCCGGATCCATGCCAGACCCGCAGGAT3’
5	Rv3852 dctd MBP RP	5’GGCCAAGCTTCTAGTGACTCGGCGCCGG 3'
6	WAG31- Sg1-For	5’- GGGATCTGGAAGTGTAGAACGGAG-3’
7	WAG31- Sg1-Rev	5’- AAACCTCCGTTCTACACTTCCAGA-3’
8	WAG31- Sg2-For	5’- GGGAGCTTACTGAACGCCACATTG-3’
9	WAG31- Sg2-Rev	5’- AAACCAATGTGGCGTTCAGTAAGC-3’
